# Phylogenetic analysis of methionine synthesis genes from *Thalassiosira pseudonana*

**DOI:** 10.1186/s40064-015-1163-8

**Published:** 2015-08-04

**Authors:** Mariusz A Bromke, Holger Hesse

**Affiliations:** Max Planck Institute of Molecular Plant Physiology, Am Mühlenberg 1, 14476 Potsdam-Golm, Germany

**Keywords:** Methionine synthesis, Diatoms, Phylogenetics

## Abstract

Diatoms are unicellular algae responsible for approximately 20% of global carbon fixation. Their evolution by secondary endocytobiosis resulted in a complex cellular structure and metabolism compared to algae with primary plastids. The sulfate assimilation and methionine synthesis pathways provide S-containing amino acids for the synthesis of proteins and a range of metabolites such as dimethylsulfoniopropionate. To obtain an insight into the localization and organization of the sulfur metabolism pathways we surveyed the genome of *Thalassiosira pseudonana*—a model organism for diatom research. We have identified and annotated genes for enzymes involved in respective pathways. Protein localization was predicted using similarities to known signal peptide motifs. We performed detailed phylogenetic analyses of enzymes involved in sulfate uptake/reduction and methionine metabolism. Moreover, we have found in up-stream sequences of studied diatoms methionine biosynthesis genes a conserved motif, which shows similarity to the Met31, a cis-motif regulating expression of methionine biosynthesis genes in yeast.

## Background

Diatoms are abundant unicellular algae in aquatic habitats. They produce massive amounts of biomass and are thought to contribute to about 20% of global carbon fixation (Falkowski et al. 1998; Field et al. 1998). Phytoplankton affects the climate on the global scale, not only by sequestration of CO_2_ but also by production of S-containing volatile compounds. According to the hypothesis proposed in 1987 by Charlson, Lovelock, Andreae and Warren (the CLAW hypothesis) phytoplankton is able to regulate the Earth’s climate through the generation of clouds by producing the precursor of dimethylsulfide (DMS). This volatile compound is oxidized in the atmosphere to give non-sea salt sulfate, which acts as cloud condensation nuclei, which in turn modifies both the degree of cloudiness and the albedo of clouds. Increased cloudiness and albedo would reduce the extent to which the surface of the planet is warmed by the sun (Charlson et al. 1987). Though, it is not clear whether the system acts as a negative feedback loop, in which higher temperature and higher availability of carbon for phytoplankton and consecutive DMS release may modulate the greenhouse effect of increased anthropogenic CO_2_ input to the atmosphere. Bacterial enzymes are mostly responsible of the formation of DMS from dimethylsulfoniopropionate (DMSP). The amino acid methionine is the precursor for the synthesis of DMSP and underlines the dependence on sulfur and nitrogen metabolism (Gage et al. 1997; Keller et al. 1999) of marine algae.

The carbon backbone of methionine derives from aspartate, which in two steps is metabolized to homoserine. In plants homoserine is activated for further processes in a reaction catalyzed by homoserine kinase (HSK), which phosphorylates the γ-hydroxyl group. In some bacteria activation of homoserine is done by *O*-succinylation catalyzed by the enzyme homoserine *O*-succinyltransferase (Zubieta et al. 2007). Phosphorylated or succinylated homoserine is condensed with cysteine by cystathionine γ-synthase to form cystathionine. Subsequently, cystathionine β-lyase hydrolyzes cystathionine to form homocysteine (Hesse and Hoefgen 2003). In fungi and bacteria *O*-acetylhomoserine and *O*-succinylhomoserine, respectively, can be directly sulfhydrylated to form homocysteine, the precursor of methionine (Foglino et al. 1995; Kerr and Flavin 1970; Park et al. 1998; Rowbury and Woods 1964). In the final step of methionine synthesis, homocysteine is methylated by two non-homologous enzymes: the cobalamin-dependent methionine synthase (METH-type) in mammals, protists and most bacteria (Evans et al. 2004; Goulding and Matthews 1997; Krungkrai et al. 1989) or the cobalamin-independent methionine synthase (METE-type) as found in bacteria (including cyanobacteria), green plants and fungi (Eichel et al. 1995; Kacprzak et al. 2003; Whitfield et al. 1970; Zeh et al. 2002). Many marine algae require external cobalamin supply to maintain methionine synthesis, though the cobalamin auxotrophy is not restricted to specific phyla (Croft et al. 2005). This suggests multiple events of METE-type enzyme loss the in the evolution of algae. Methionine not only is a proteogenic amino acid but served a substrate for synthesis of many sulfur-containing metabolites, such as a very important cofactor in methylation reactions, S-adenosylmethionine (SAM) (McQueney et al. 2000).

Research on diatoms advanced significantly with publication of the whole genome sequences of *Thalassiosira pseudonana* (Armbrust et al. 2004) and *Phaeodactylum tricornutum* (Bowler et al. 2008). Diatoms are amazing organisms derived from a non-photosynthetic eukaryote that domesticated a photoautotrophic eukaryotic cell phylogenetically close to a red alga (Parker et al. 2008). This resulted in an extensive gene transfer events and genomic reorganization. Secondary endocytobiosis also increased the complexity of diatom cell structure, with implications on physiology and biochemistry. An important aspect of diatom cell morphology is that plastids are surrounded by four rather than two membranes with the consequence that all nuclear-encoded plastid proteins have to cross four membranes (Gibbs 1979). The same is true for the exchange of metabolites between plastids and the cytoplasm. There is still little knowledge on the sulfur uptake and assimilation (Bromke and Hesse 2013; Kopriva et al. 2008). To shed more light on the biosynthesis of methionine in the model diatom *T.pseudonana*, we have performed a detailed phylogenetic analysis of each enzyme from the methionine biosynthesis pathway in this organism. In addition, we have addressed a subcellular localization question in order to present possible models of the pathway.

The compartmentation of sulfate assimilation and methionine synthesis is an intriguing aspect in understanding S-assimilation and methionine synthesis. While sulfate activation takes place in the cytosol and in plastids, OAS and cysteine are synthesized in all three compartments: cytosol, plastid, and mitochondria (Hesse et al. 2004; Krueger et al. 2009). In photosynthetic eukaryotes, sulfate reduction is confined to plastids (Brunold and Schiff 1976; Hesse et al. 2004), the same as the synthesis of cystathionine and homocysteine. *Arabidopsis* contains three functional isoforms of the cobalamin-independent (CIMS) enzyme. One of them is located in chloroplasts and is devoted to the de novo synthesis of methionine. The two other methionine synthase isoforms in *Arabidopsis* are located in the cytosol and most probably are responsible for the regeneration of methionine by re-methylation of homocysteine (Ravanel et al. 2004).

To shed more light on the structure of methionine synthesis genes in *T.pseudonana* as well as to put them in the evolutionary context, we have performed a phylogenetic analysis on eight sequences. These sequences and additional enzyme sequences from homoserine and glutathione synthesis pathways were analyzed to investigate their subcellular localization.

## Results and discussion

### Identification of methionine biosynthesis pathway proteins in *Thalassiosira pseudonana*

Methionine belongs to the aspartate-family of amino acids. Hence, we have taken for analysis not only enzymes of sulfur transfer and methionine synthesis, but also enzymes responsible for the synthesis of methionine’s carbon backbone from aspartic acid. In this study plant, bacterial and fungal genes known to be involved in the methionine biosynthesis pathway were used as queries to identify homologous genes in the genome of *T. pseudonana*. The sequences were analyzed to predict subcellular localization of the proteins. Additionally to the methionine synthesis genes, we have analyzed the localization of two enzymes of the glutathione synthesis pathway. Identified genes with predicted localization of their products are presented in Table 1, which gives additional information about used abbreviations and database entry numbers (http://genome.jgi-psf.org/Thaps3/Thaps3.home.html) as well.

**Table 1:** Predicted target peptides of selected proteins of *Thalassiosira* *pseudonana*

Protein	Protein ID	Length	Loc	RC	Mito	Secr	Other	TP length	Cleavage site	CD start
Aspartate kinase	29008	936	S	5	0.437	0.54	0.034	19	VTS-FAP	90
Aspartate kinase	269371	619	S	1	0.033	0.887	0.078	26	IGA-FSP	144
Aspartate semialdehyde dehydrogenase	32577	345	_	2	0.074	0.121	0.859	–		6
Homoserine dehydrogenase	40655	435	_	1	0.048	0.096	0.926	–		5
Homoserine dehydrogenase	24587	502	_	4	0.271	0.15	0.552	–		128
Homoserine dehydrogenase	29144	433	_	3	0.249	0.045	0.775	–		1
Homoserine acetyltransferase	12141	375	_	1	0.081	0.062	0.938	–		65
Homoserine kinase	28592	358	M	5	0.439	0.082	0.412	36	VPA-SSA	28
Cystathionine β-lyase-like	269520	456	M	5	0.49	0.065	0.459	66	SAH-DGS	1
Cystathionine γ-synthase-like	9830	381	_	1	0.037	0.114	0.95	–		61
Methionine γ-lyase-like	11993	487	M	3	0.765	0.025	0.257	39	ENK-HAT	45
Methionine synthase	693	1,359	_	2	0.123	0.066	0.878	–		85
SAM synthetase	21815	465	_	2	0.098	0.076	0.886	–		4
SAM synthetase	39946	450	_	3	0.247	0.059	0.741	–		65
Glutamylcysteine synthetase	13064	636	M	5	0.57	0.053	0.445	36	NRV-KDL	231
Glutathione synthetase	29212	533	_	3	0.044	0.298	0.826	–		6

The first two columns “ProteinID” and “Length” contain entry numbers of protein models and its amino acids length. In the “Loc” column is the predicted localization: *S* secreted, *M* mitochondrial, _ any other localization. *RC* stands for Reliability Class of the prediction (scale: 1 to 5, where 1 is the best). Columns titled “mito”, “secr”, “other” contain final NN scores on which the prediction is based for mitochondrial, secretion and other localization; respectively. The “TP length” and “cleavage site” columns present predicted presequence lengths and the respective cleavage site motif. The “CD Start” column contains lengths of N-terminal sequences preceding Conserved Catalytical Domains of respective proteins.

A Maximum Likelihood algorithm implemented in the PhyML software was used to investigate phylogenetic relationships between *T. pseudonana* protein sequences and their eukaryotic and prokaryotic homologues. A limited species number as representatives of different phyla was selected (listed in Methods). For generation of phylogenetic trees homoserine acetyltransferase, cystathionine γ/β-synthase-like proteins, methionine synthase, and S-adenosylmethionine synthetase orthologues were used. In case of pyridoxal phosphate-dependent enzymes (cystathionine metabolism enzymes) the respective phylogenetic tree was used to predict enzymatic activity of analyzed proteins.

### Prediction of intracellular localization

On the basis of the obtained amino acid sequences, the subcellular localization of proteins involved in sulfur assimilation and methionine synthesis was predicted. In the C-terminal parts of analyzed proteins no motifs responsible for retention in the endoplasmic reticulum (KDEL, DEEL or DDEL) were found. Location assignment was performed with TargetP software and it is based on the predicted presence of N-terminal mitochondrial targeting peptide (mTP) or secretory pathway signal peptide (SP). Additionally, the protein sequences were analyzed for any N-terminal sequences longer than 15 amino acids in front of conserved catalytic domains. These pre-sequences might form specific diatomal targeting peptides, which are not recognized by the used software. The results of the prediction are presented in Table 1. The prediction reliability values are a scale from 1 to 5, where 1 indicates the strongest prediction.

Out of analyzed sequences six show a pre-sequence that could be used by the cellular machinery to traffic the proteins to the respective compartment. In two aspartate kinase genes we have detected sequences which according to the used software, would direct these proteins into secretion via endoplasmic reticulum. Due to the evolutionary history of diatoms, the cells of these organisms contain plastids surrounded by additional two layers of plasma membrane, from which the outermost membrane is continuous with the endoplasmic reticulum (Gibbs 1979). The result suggests that these enzymes are directed to the plastid. In fact, we have detected in both aspartate kinase sequences N-terminal bipartite pre-sequences, which consist of a signal and a transit peptide-like domain responsible for directing the nuclear-encoded protein into diatomal chloroplast. As demonstrated experimentally by means of a heterogeneous protein expression system in diatom *P. tricornutum*, presence of a conserved amino acid motif at the SP cleavage site (ASA-FAP) is associated with plastid-localized proteins (Gruber et al. 2007; Kilian and Kroth 2005). The aspartate kinase sequences do not have the exact ASA-FAP sequence, but contain phenylalanine in the 4th position of the predicted cleavage motif. It has been shown that phenylalanine in this position is crucial for successful transfer to the plastid. In some cases phenylalanine (F in the motif sequence) can be exchanged by other bulky amino acid—tryptophan, tyrosine or leucine (Gruber et al. 2007). Phenylalanine and valine were found in pre-sequences of two genes of the sulfur reduction pathway in diatoms (Bromke and Hesse 2013). One should note that this process in phototrophs takes place exclusively in plastids.

There are four sequences that are predicted to be directed into mitochondria: cystathionine β-lyase-like, homoserine kinase, γ-glutamylcysteine synthetase and methionine γ-lyase-like (Table 1). The prediction reliability values are as low as 5, with an exception for methionine γ-lyase-like, in which case the prediction reaches the class 3. In plants reactions of homoserine phosphorylation by homoserine kinase as well as the downstream synthesis of cystathionine and homocysteine are localized to plastids (Hesse and Hoefgen 2008), whereas these processes take place in the cytosol of fungi (Marzluf 1997). Moreover, methionine-γ-lyase in *Arabidopsis thaliana* is a cytosolic enzyme (Rebeille et al. 2006). Thus, the prediction of localization of these proteins seems not to be accurate. In animals and fungi γ-glutamylcysteine synthetase and glutathione synthetase gene products are localized to the cytosol (Griffith 1999; Pócsi et al. 2004). In plants, due to the transcript heterogeneity, the synthesis of the glutathione can take place in chloroplast and in cytosol depending on the length of the respective gene transcript (Wachter et al. 2005). Database searches revealed the presence of putative TP sequences for maize γ-glutamylcysteine synthetase and glutathione synthetase proteins (Wachter et al. 2005), despite absence of plastidic TP in cDNA clones reported by Gómez et al. (2004). There is only one sequence of γ-glutamylcysteine synthetase in the genome of *T. pseudonana*, but it is incomplete as it lacks a starting ATG codon. The gene model of γ-glutamylcysteine synthetase shows high similarity to heteroconts and less to animal sequences. Taking this into consideration, we suggest that the enzyme is localized to the cytosol although TargetP predicts a mitochondrial localization (weak, class 5 prediction). On the other hand, the analysis of the identified glutathione synthetase sequence resulted in a strong score suggesting its cytosolic location of the enzyme. Thus, the glutathione synthesis takes place in the cytosol and is subsequently transported to other cell compartments for biochemical functions.

### Analysis of phylogenetic relationship of *Thalassiosira pseudonana* genes

#### Phylogenetic analysis of enzymes involved in the transacetylation of homoserine

**Fig. 1:**
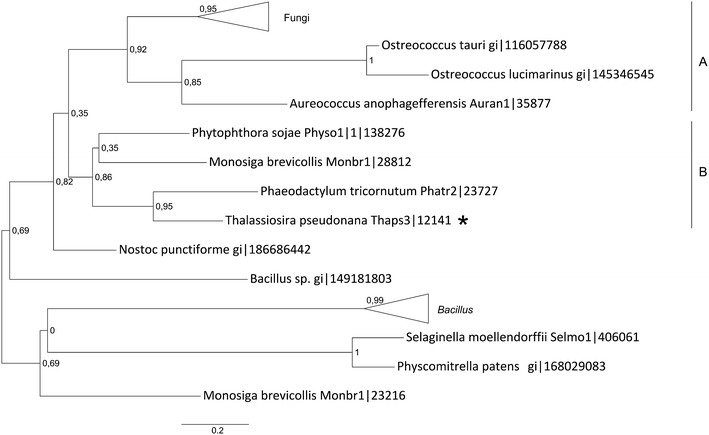
Phylogenetic tree of homoserine acetyltransferase proteins. *T. pseudonana* protein is marked with an *asterisk*. The phylogenetic tree was reconstructed using the maximum likelihood method implemented in the PhyML program (curated alignment with 105 characters was used). The *T. pseudonana* protein is marked with an *asterisk*. Reliability for internal branch was assessed using the aLRT. The *number* next to species name represents entry code of given protein in a databank.

An interesting feature of *T. pseudonana* genome is that it contains next to the identified homoserine kinase a gene encoding a protein with high similarity to homoserine acetyltransferase (HAT; Thaps3: 12141). This is interesting as finding as in some bacteria (Park et al. 1998) and fungi (Kerr and Flavin 1970) the activation of homoserine depends on transacetylation—formation of *O*-acetylhomoserine using acetyl-CoA as substrate. According to the prediction (Table 1), HAT in cells of *T. pseudonana* is cytosolic localized. The phylogenetic tree inferred for the enzyme shows that the putative homoserine acetyltransferase of *T. pseudonana* can be found in one cluster (Fig. 1, cluster B) together with predicted protein sequences from diatom *P. tricornutum*, oomycete *Phythophora sojae* (both heterokonts) and a choanoflagellate *Monosiga brevicolis* (a metazoan). The exact topology of this cluster is not resolved. Although, a close relationship between diatomal proteins and the HAT from *M. brevicolis* was reported previously (Sun et al. 2010). These also seem to be related with homoserine acetyltransferases from fungi (Fig. 1, cluster A), in which the direct sulfhydrylation of *O*-acetylhomoserine was experimentally confirmed (Wiebers and Garner 1967). *O*-acetylhomoserine (OAH) is also used by bacterial and cyanobacterial cells to synthesize homocysteine in the process of sulfhydrylation, in which sulfide is bound with OAH yielding homocysteine (Hwang et al. 2002; Kerr 1971). It is likely that *T. pseudonana* is able to perform a similar reaction in order to synthesize homocysteine from *O*-acetylhomoserine. If this is the case, *O*-phosphohomoserine (OPH) would function mainly as substrate for threonine synthesis as has been shown for bacteria and fungi (Chassagnole et al. 2001; Farfan and Calderon 2000) and not, as in plants, for the synthesis of cystathionine (Curien et al. 1996, 1998; Zeh et al. 2001). This hypothesis has to be verified experimentally.

#### Phylogenetic analysis of enzymes involved in the phosphorylation of homoserine

The activation of homoserine for synthesis of homocysteine can be done by the phosphorylation of the gamma-hydroxyl group as well. This reaction is catalyzed by the special enzyme homoserine kinase (HSK). The *T. pseudonana* genome contains only one copy of the HSK (Thaps3: 28592). In the genome of *Cyanidioschyzon merolae,* the red algae model organisms, no homoserine kinase gene model could be found. Nevertheless, the genome of *C. merolae* contains genes annotated as aspartate kinase/homoserine dehydrogenase as well as threonine synthase—enzymes producing substrate for HSK and using *O*-phosphohomoserine (OPH). The synthesis of OPH is the only way of homoserine activation in plants and green algae (Hesse and Hoefgen 2003).

**Fig. 2:**
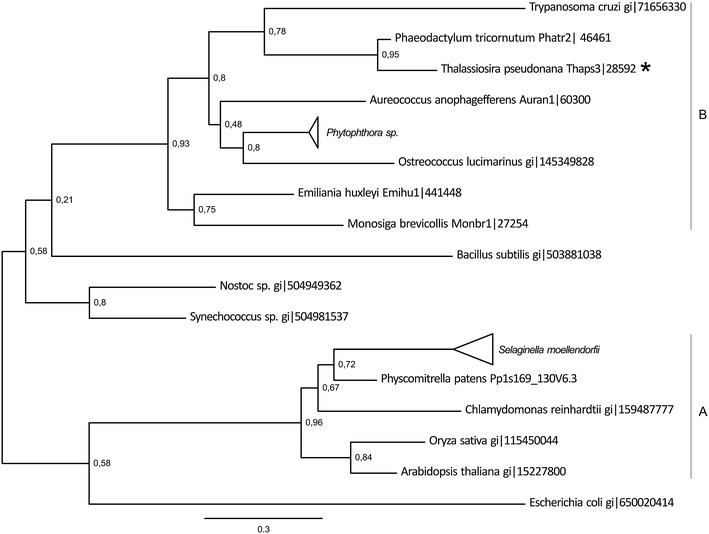
Phylogenetic tree of homoserine kinase proteins (HSK). The phylogenetic tree was reconstructed using the maximum likelihood method implemented in the PhyML program (curated alignment with 99 characters was used). The *T. pseudonana* protein is marked with an asterisk. Reliability for internal branch was assessed using the aLRT. The *number* next to species name represents entry code of given protein in a databank. Collapsed branches contain sequences of the same species or genus.

This fact is visible on the phylogenetic tree generated from HSK sequences. All plant proteins cluster together (Fig. 2a). The HSK from *T. pseudonana* is found in one cluster with other diatomal sequence and the one from *Trypanosoma cruzi* (Fig. 2b). This observation might suggest that the HSK in diatoms is evolutionary distant from the plant isoform and thus the OPH and threonine biosynthesis in diatoms is probably differently regulated. Unlike in plants where OPH is the branching point between biosynthesis pathways of methionine and threonine, the *T. pseudonana* HSK might be delivering the substrate for Thr synthesis and is competing for homoserine with HAT. Other sequences from oomycetes and heterokonts can be found in the cluster B (Fig. 2), though its final topology could not be defined.

#### Phylogenetic analysis of enzymes catalyzing the formation of methionine precursors

Due to their high similarity it is impossible to distinguish cystathionine γ/β-synthases from cystathionine β/γ-lyases solely on basis of their sequences. Both types of enzymes utilize pyridoxal 5′-phosphate (PLP) as cofactor in reactions of cystathionine synthesis and/or lysis. A similar catalytic domain can be found in methionine γ-lyase (MgL), also a PLP-dependent enzyme. MgL is a promiscuous enzyme, which catalyses a number of α/γ- and α/β-elimination reactions on methionine and cysteine derivatives. It also catalyses the γ-replacement reaction of the thiomethyl group of substrates such as methionine, homocysteine and ethionine to yield the corresponding S-substituted homocysteine derivative (Lockwood and Coombs 1991). The enzyme has been found and characterized in various bacteria (Dias and Weimer 1998; Faleev et al. 1996; Inoue et al. 1995; Manukhov et al. 2005), protozoans like *Trichomonasvaginalis* (McKie et al. 1998), and *Entamoebahistolytica* (Tokoro et al. 2003), and in *Arabidopsis* (Rebeille et al. 2006). The predicted plant protein has 28–32% identity (48% similarity) with methionine γ-lyase from *Pseudomonas putida* and *E.histolytica*, respectively (Rebeille et al. 2006).

**Fig. 3:**
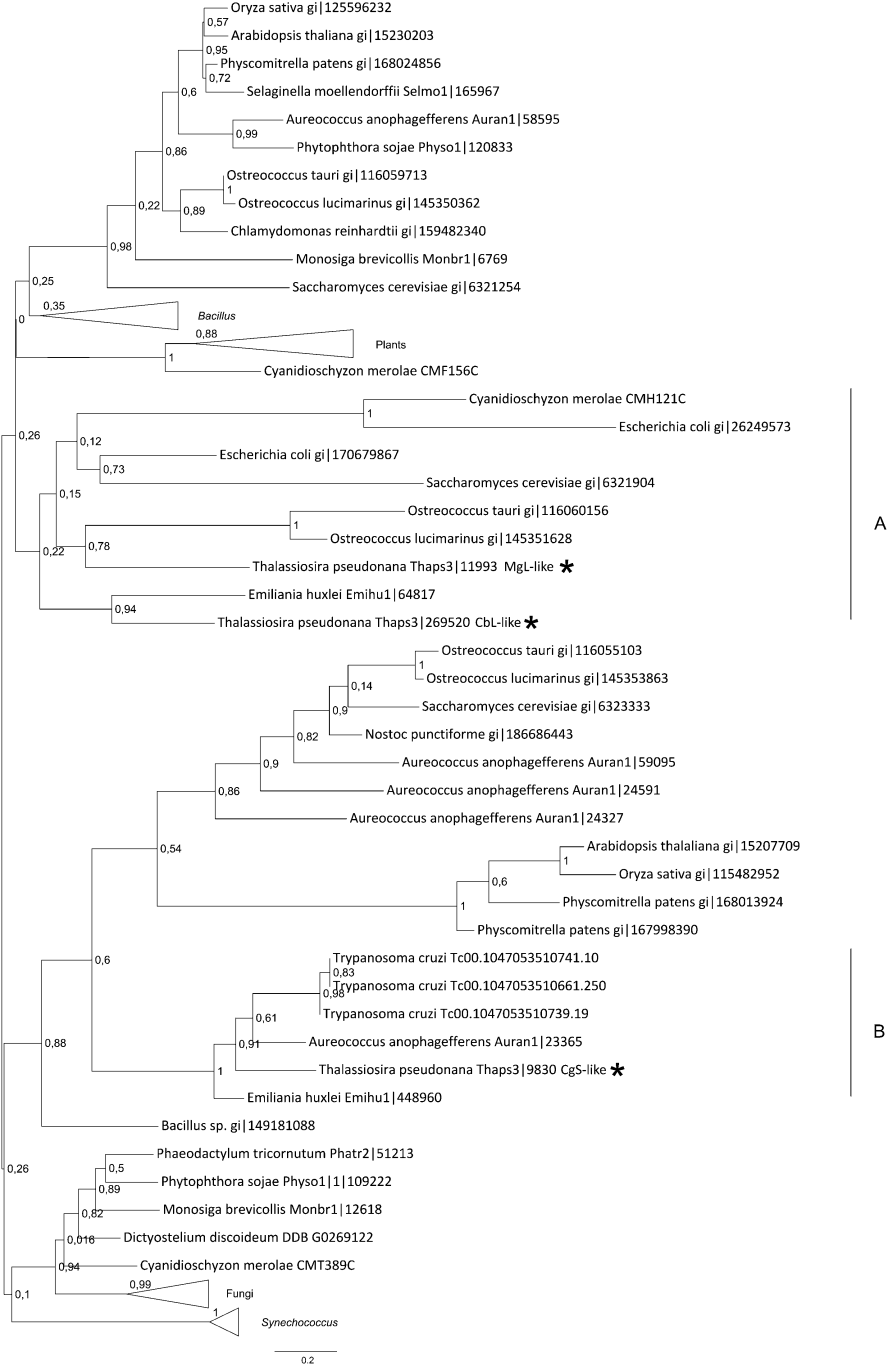
Phylogenetic tree of PLP-dependent enzymes. The phylogenetic tree was reconstructed using the maximum likelihood method implemented in the PhyML program (curated alignment with 149 characters was used). *T. pseudonana* proteins are marked with *asterisks*. Reliability for internal branch was assessed using the aLRT test. The *number* next to species’ name represents entry code of given protein in a databank.

Therefore, three sequences found in the genome of *T. pseudonana*—cystathionine synthase-like (CgS-like; Thaps3: 9830), cystathionine lyase-like (CbL-like; Thaps3: 269520) and methionine γ-lyase-like (MgL-like; Thaps3: 11993)—were analyzed together (Fig. 3). The cystathionine synthase-like protein clusters together with proteins from the marine algae coccolitophorid *Emiliania huxlei,* the harmful algal blooms-causing *Aureococcus anophagefferens* and the parasitic eukaryote *T. cruzi* (Fig. 3). Protozoa, such as *Trypanosoma,* synthesize cysteine from methionine (Walker and Barrett 1997), which suggests that this diatomal enzyme might catalyze the same reaction and therefore could be described as putative cystathionine γ-lyase. Still, without biochemical characterization, there are no means to correctly predict whether the enzyme functions as cystathionine synthase or lyase. The methionine γ–lyase-like protein (MgL-like) and the putative cystathionine lyase (CbL-like) from *T. pseudonana* are found in one cluster together with proteins from marine eukaryotic algae, *Escherichia coli* and yeast (Fig. 3, cluster A). The whole clade is very weakly supported (aLRT = 0.26). Little is known about the catalytic character of the enzymes from photosynthesizing algae. One can assume that green algae *Ostreococcus* catalyze synthesis of homocysteine from cystathionine in the plant manner (other sequences of *Ostreococcus* proteins are found neighboring with plant enzymes suggesting conservation) and thus, the MgL-like enzyme from *T. pseudonana* (Thaps3: 11993) would catalyzes the same reaction. The third analyzed PLP-dependent enzyme from the diatom (Thaps3: 269520) clusters together with a sequence from *E. huxlei.* Its function remains unknown. Noteworthy, the analyzed diatomal proteins do not form common clusters with plant-derived sequences (Fig. 3).

#### Phylogenetic analysis of methionine synthase and SAM synthetases

**Fig. 4:**
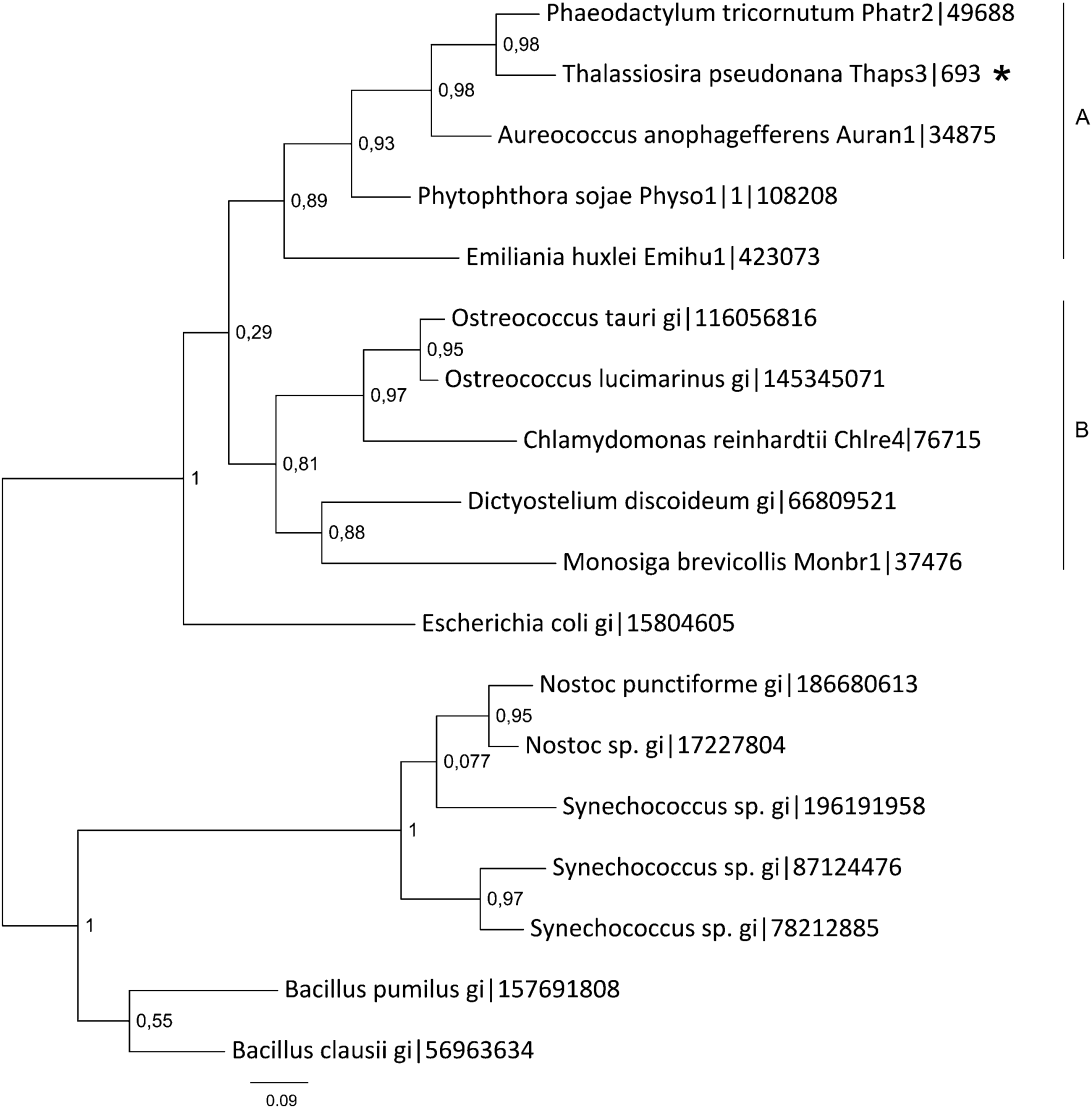
Phylogenetic tree of methionine synthase proteins. The phylogenetic tree was reconstructed using the maximum likelihood method implemented in the PhyML program (curated alignment with 811 characters was used). The *T. pseudonana* protein is marked with an *asterisk*. Reliability for internal branch was assessed using the aLRT. The *number* next to species’ name represents entry code of given protein in a databank.

Methionine synthase catalyzes the reaction of transmetylation, where a methyl group is transferred from 5′-methyltetrahydropteroyl glutamates onto the sulfur atom of homocysteine. Methylation of homocysteine can be catalyzed by two non-homologous enzymes: a cobalamin-dependent (METH) and cobalamin-independent methionine synthase (METE). Vitamin B_12_ (cobalamin) is not needed for the enzymatic reaction catalyzed by enzymes from some bacteria, plants and fungi (Eichel et al. 1995; Kacprzak et al. 2003; Whitfield et al. 1970; Zeh et al. 2002). Cobalamin-dependent methionine synthase is found in mammals, protists and most bacteria (Evans et al. 2004; Goulding and Matthews 1997; Krungkrai et al. 1989). Both enzymes share no sequence similarity. In the genome of *T. pseudonana* only one sequence was found which could be assigned as encoding a methionine synthase enzyme. Analysis of the sequence showed similarity to the eukaryotic METH-type of the enzyme and thus is most likely localized to the cytosol. This is also supported by the TargetP analysis (Table 1). The phylogenetic analysis of cobalamin-dependent methionine synthase proteins shows two main clusters (Fig. 4). The smaller cluster contains cyanobacterial methionine synthases and sequences of proteins from two *Bacillus* species. The other cluster contains sequences of enzymes from diverse organisms—eukaryotes (Fig. 4, clusters A and B) with one prokaryotic exception—MetH from *E. coli*. Methionine synthase form *T. pseudonana* is found in clade A (Fig. 4) with homologues from *P. tricornutum, A. anophagefferens* and *Phytophthora sojae*. These four organisms share common ancestor (Parker et al. 2008). Noteworthy, in the genome of other diatom *P. tricornutum* a long methionine synthase gene could be found (Phatr2, ProteinID 23399). This gene model contains a 2,280-long intron, which in the same reading frame as both exons encodes a conserved domain of MetH and pterin-binding domain. This is probably a result of a mutation-duplication which led to doubling of the domain. The *P. tricornutum* genome repository at Joint Genome Institute (http://genome.jgi-psf.org/Phatr2) contains also EST’s of the intron suggesting the presence the intron-bearing mRNA in cells and validates the genome sequencing outcome. The presence of two MetH domains has to be verified experimentally. Moreover, the organism contains also a METE-type methionine synthase, which shows similarity to prokaryotic cobalamin-independent enzymes and to the one found in *Chlamydomonas reinhardtii*. The clade B contains MS sequences from green algae and primitive eukaryotes—choanoflagellate *Monosiga brevicollis* and amoeba *Dictyostelium dyscoideum* (Fig. 4, cluster B). We can conclude from the obtained data that cobalamin-dependent MS of *T. pseudonana* might originate from an enzyme of the non-photosynthetic eukaryotic ancestor, while the METE-type of enzyme coming from the genome of a phototrophic eukaryote (ancestor of the diatomal plastid) was lost during evolution.

**Fig. 5:**
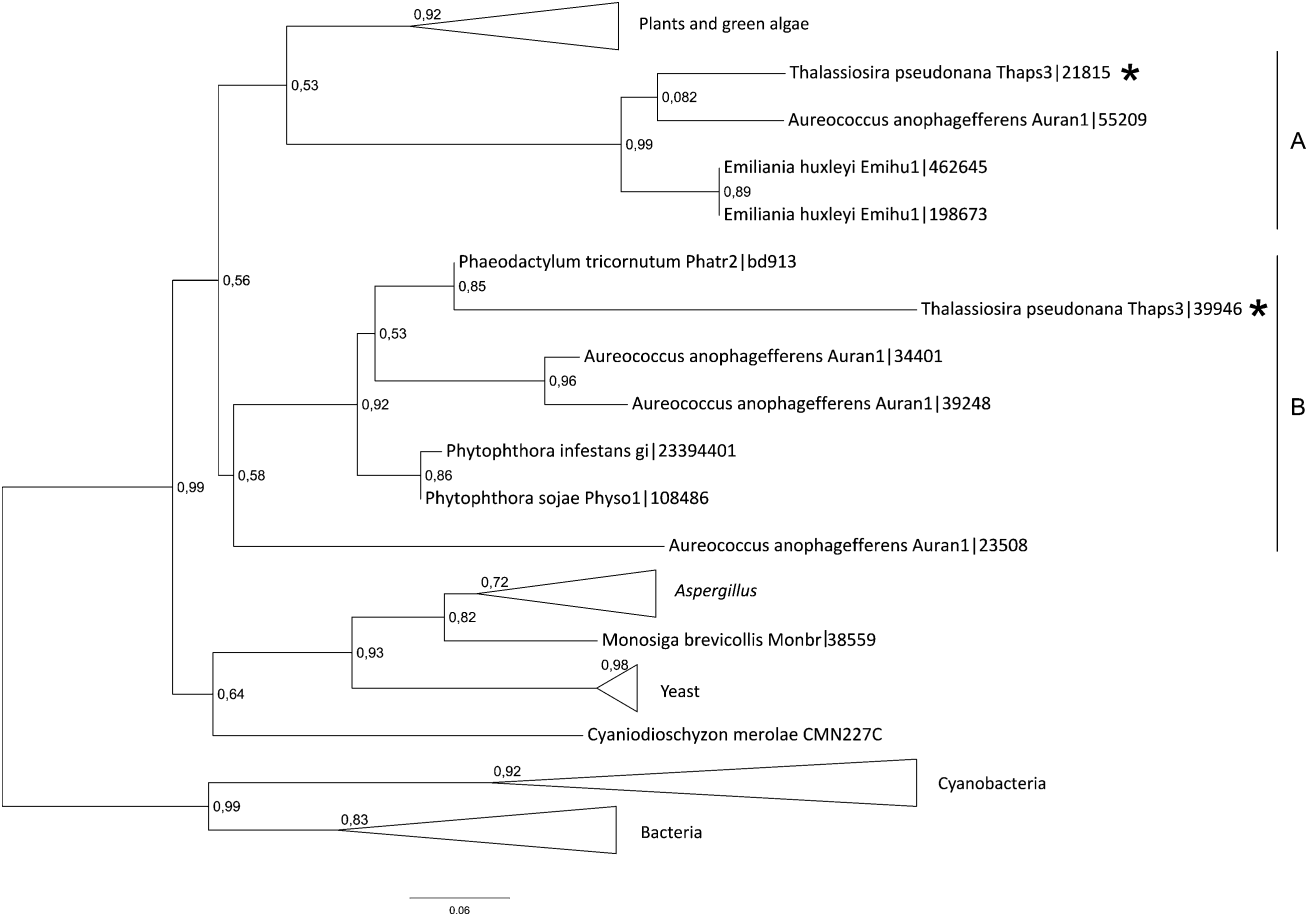
Phylogenetic tree of S-adenosylmethionine proteins. The phylogenetic tree was reconstructed using the maximum likelihood method implemented in the PhyML program (curated alignment with 315 characters was used). *T. pseudonana* proteins are marked with *asterisks*. Reliability for internal branch was assessed using the aLRT test. The *number* next to species’ name represents entry code of given protein in a respective databank.

S-adenosylmethionine synthetase (SAMS) catalyzes the reaction forming SAM from methionine and ATP. Multiple S-adenosylmethionine synthetase isoenzymes can be found in divergent organisms. Single isoforms of SAM synthetase exist in *Plasmodiumfalciparum* (Chiang et al. 1999) and in *Drosophilamelanogaster* (Larsson and Rasmuson-Lestander 1997). Higher plants like *Cantharanthus roseus* have three isoforms of SAMS (Schröder et al. 1997), whereas in the rice genome two genes could be identified encoding SAMS (Lee et al. 1997). Despite evolutionary divergence, the SAMS enzyme is higher conserved. Between species approximately 30% of S-adenosylmethionine proteins are identical in almost every species (Sánchez-Pérez et al. 2004). The genome of *T. pseudonana* contains two sequences of S-adenosylmethionine synthetase. The absence of a predictable SP at the N-terminal part of both SAM synthetase isoforms suggests that these enzymes are located in the cytosol. The phylogenetic tree of SAMS sequences shows separation of eukaryotic an prokaryotic enzymes (Fig. 5). SAM synthetases from *T. pseudonana* are found in the clusters A and B (Fig. 5). The first cluster, apart from SAMS1 (Thaps3:21815) contains two *E. huxleyi* sequences and one *A. anophagefferens*. The second cluster (Fig. 5, cluster B) with SAMS2 (Thaps3:39946) contains sequences from organisms belonging to the *Heterokonthophyta* clade. Fungal and plant sequences are found in well defined clusters. The sequence comparison suggest that diatomal SAM synthases might originate from two lineages of organisms, which reflects the history of diatoms (Parker et al. 2008): SAM synthetase 2 originates from the heterotrophic host (Thaps3:39946) while the ancestor of SAMS1 derives from the engulfed phototrophic algal cell (Thaps3:21815).

### Analysis of the up-stream DNA sequences of methionine metabolism genes

**Fig. 6:**
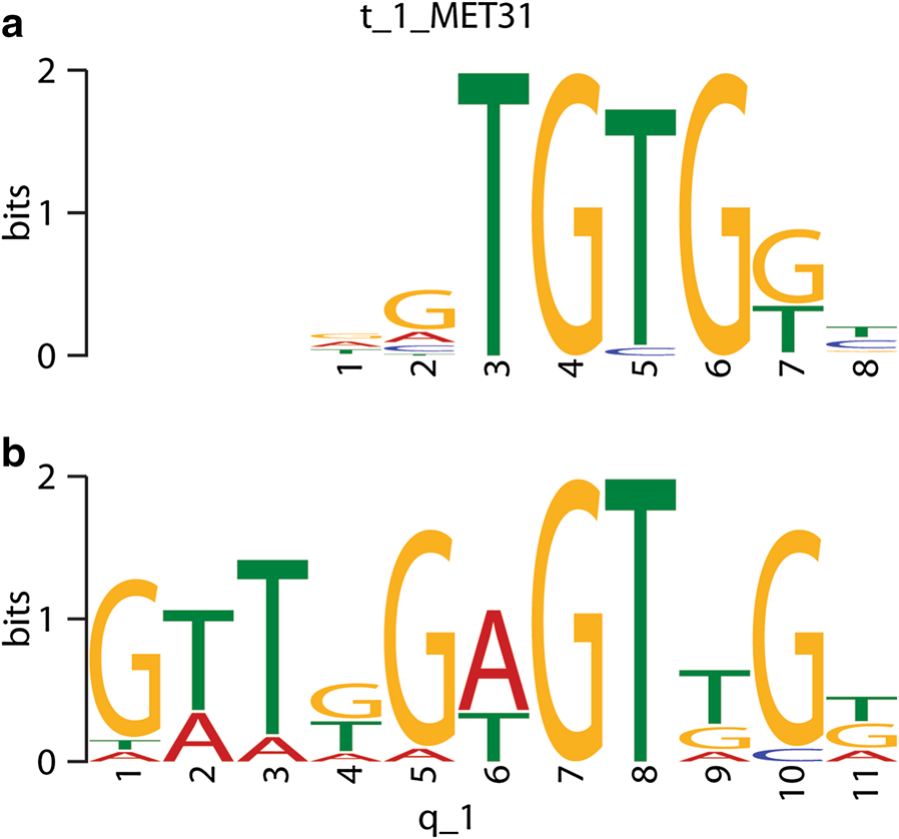
The sequence-logo comparison of conserved motifs between **a** yeast MET31 binding site and **b** the motif found in putative promoters of *T. pseudonana* methionine metabolism genes.

To gain insight on the regulation of the gene expression we have analyzed DNA sequences of putative promoter regions of the methionine metabolism genes. We have compared sequences of 16 genes in search for conserved motifs. Additionally a set of ten up-stream sequences of ten genes not-related with sulfur amino acids metabolism was used as a negative control. The analysis resulted in three conserved sites present in the pool of studied sequences and absent in the negative control set (see Methods chapter). The best result was a 11-nt-long motif G[TA]T[GT]G[AT]GT[TG]G[GTA] (consensus sequence with variable nucleotides in square brackets), which was found in 15 out of analyzed 16 sequences (Table 2). The E value that is the measure of statistical significance calculated on motifs log likelihood ratio, its width, number of sites and the background letter frequencies in the training set. For the best match discussed here the E value was 4.2e−3. Subsequently the motif was compared using TOMTOM with a database of prokaryotic, yeast and plant motifs. Matching with plants and prokaryotic motifs produced ambiguous results, where as the comparison with the collection of yeast cis-regulatory motifs resulted in identification of a MET31 binding site (p value 0.0487645 and E value 5.9724) (Fig. 6). Only one sequence from the analyzed pool that didn’t contained said motif belonged to one of two apartate kinases. The phosphorylation of aspartate is a regulatory point of the whole methionine and threonine synthesis pathway in yeast (Ramos et al. 1991) and plants (Curien et al. 2008). These data can be interpreted that at least one isoform is differently regulated and does not belong to the Met/Thr-regulatory network.

**Table 2:** Predicted conserved motifs in 15 putative promoters of *T.* *pseudonana* genes

Gene	Protein ID	Length	Start	*p* value	Sites		
Aspartate kinase	269371	500	232	1.31e−05	GATGCTCACG		TCTCGCCATG
Aspartate semialdehyde dehydrogenase	32577	500	114	5.43e−05	GGCCATTGTG		GGTAAAAAAG
Cystathionine β-lyase-like	269520	1,000	780	1.32e−07	GGAGAAGAGG		AAGGTCGTGT
Cystathionine γ-synthase-like	9830	500	45	3.82e−05	GAGGGCGGAG		GCCCTCTCTC
Homoserine acetyltransferase	12141	700	64	8.59e−06	AAGAGGCTTC		CGACGACAGA
Homoserine dehydrogenase	29144	300	60	4.12e−05	ACAACTGAAC		CTTGGTGTGC
Homoserine dehydrogenase	40655	500	332	1.72e−06	TCCTTTGCGT		CTTGATGTAT
Homoserine dehydrogenase	24587	300	154	3.92e−06	GTTAGTTGTT		TTGGTTTGCT
Homoserine kinase	28592	1,000	81	2.85e−07	CCATATCTTT		TTTCGATCGA
Methionine synthase	693	1,000	36	4.39e−07	CGTCGACAGA		CTGAAACAGA
Methionine γ-lyase-like	11993	700	323	6.40e−06	GCGTTGACAT		GCCTCATGTC
SAM synthetase	21815	500	336	9.81e−06	AACGTGCCTC		CTGGTATGGT
SAM synthetase	39946	300	152	1.04e−05	ACCATTGTTT		GGGTAACAGC
Glutamylcysteine synthetase	13064	1,000	4	3.29e−05	GTG		TGCTTTTCGG
Glutathione synthetase	29212	1,000	700	3.41e−05	GCTTCATGAG		GTGTTTGGAG

The columns “Protein ID” and “Length” contain entry numbers of protein models and length of is DNA sequence up-stream of 5′-UTR. The following columns present start position, random letters probability and the sequence of the conserved motif with ten nucleotide padding.

Presence of different transcription controllers enables integration of various stimuli and proper response on the level of gene expression. This includes binding of different transcription factors to different DNA-binding sites within a promoter and binding of different members of a transcription factors family to one site in a promoter. In budding yeast, *Saccharomycescerevisiae,* organic sulfur metabolism is regulated by the Met4 transcriptional system (Lee et al. 2010). The Met4 cannot bind to DNA but through protein–protein interaction with either of two highly similar DNA-binding proteins, Met31p and Met32p, or with a homodimer of Cbf1 proteins. Met31p and Met32p are C2H2-type zinc finger proteins which bind to consensus motif AAACTGTGG found in 5′-upstream of several genes of the sulfur metabolism in yeast (Blaiseau et al. 1997). The Cbf1 (centromere-binding factor 1) is a basic helix-loop-helix protein, which forms homodimers to bind to sites with consensus CACGTG core present at MET gene promoters (Kuras et al. 1996). This makes it to one of main activators of methionine metabolism and glutathione synthesis genes in yeast. Very little is known about the transcriptional regulation of gene expression in diatoms (Upper 1974). In genomes of *T. pseudonana* and *P. tricornutum* as much as 46 and 40 zinc finger transcription factors were identified, respectively. These belong to three types: C2H2, CCCH and TAZ type (Rayko et al. 2010). Neither, Met4p nor Met31p/Met32p yeast orthologs were found in the genome of *T. pseudonana.* Though, it can not be excluded that the regulation of the sulfur metabolism in this diatom might rely on similar conserved mechanism, including conserved protein binding sites in the promoter regions of the involved genes, as in unicellular organisms like yeast.

## Conclusions

Our goal in this study was to identify core features enabling to predict potential exchanges between species and localisation of proteins in order to get insight into the localization of cysteine and methionine pathways in diatoms. Due to the fact that relatively few sequences are known from diatom species, current subcellular localization prediction algorithms are not capable to assign some of the tested protein sequences to their compartments. In most cases it was possible to identify the signal and transit peptide-like domains in sequences of proteins localized to plastids, which was based on the previous observations made in other organisms. However, in other cases the prediction of the localization of proteins known to be targeted to plastids was not possible, which might be caused by modified and/or adapted signal peptides allowing crossing four membranes into the lumen of plastids.

The analysis of gene sequences encoding enzymes of the methionine synthesis pathway revealed that the predicted proteins are specific for algae of the chromalveoates or heterokont type. The homology of the tested proteins drops down comparing the algae proteins with orthologous proteins from plants and even more when compared to bacterial or cyanobacterial proteins. In consequence, the results mirror the distinct evolutionary path of diatoms.

The homology search made it possible to identify two enzymes, HSK and HAT, utilizing homoserine as precursor to direct this compound into pathways forming either threonine or homocysteine, respectively. The analysis does not allow to rule out that similar to yeast acetylated homoserine is the acceptor for reduced sulfur from sulfur assimilation pathway. To proof this finding and specify the true catalytic properties of the PLP-enzymes (CbL-like, CgS-like, and MgL-like) further biochemical studies have to be done. Moreover, the identification of a common motif in promoter regions of 15 genes involved in methionine metabolism of *T. pseudonana* suggests evolutionary conservation of the regulatory mechanism of the pathway. This finding, together with the homoserine acetyltransferase gene supports the hypothesis of the residual “fungal-type” of sulfur amino acids biosynthesis pathway in diatoms (Bromke and Hesse 2013). Phylogenetic consideration revealed that in *T. pseudonana* methionine synthase represents the MetH type of identified methionine synthases requiring cobalamin as cofactor. The cobalamin auxotrophy in *T. pseudonana* is probably the basis for a symbiotic relationship with cobalamin-producing bacteria.

The intensive analysis of the N-terminal site of methionine synthase protein could not identify a transit peptide and thus the result strongly suggests that the synthesis of methionine takes place in the cytosol, which would be in concordance with methionine synthesis in plants. It is likely that during the evolution *T. pseudonana* lost its cobalamin-independent enzyme. On the other hand SAM synthetase 1 seems to be retained from the phototrophic ancestor algae, whereas the SAMS 2 is a legacy of the heterotrophic ancestor-host cell.

Diatoms are fascinating organisms and studying their biology will bring many interesting observations and conclusions. Sequencing more and different diatoms will help increase our knowledge and to refine the prediction algorithms in order to get insight into diatoms metabolism.

## Methods

### Sequence selection

Amino acid sequences of annotated sulfur metabolism gene models of *T. pseudonana* were used as queries to perform protein–protein BLAST (Altschul et al. 1997) in order to identify the orthologous genes from other organisms. The search was restricted to a set of organisms representing different prokaryotic and eukaryotic organisms: *E. coli, Bacillus sp., Synechococcus sp., Nostoc sp., P. tricornutum, E. huxlei, A. anophagefferens, Ostreococcus* sp.*, C. reinhardtii, Phytophthora sp, Selaginella moelendorffi, Physcomitrella patens, A. thaliana, Oryza sativa, Porphyra purpurea, C. merolae, Monosiga brevicollis, Dictyostelium discoideum,* and *T. cruzi*. Hit-results with E value >1e−10 were discarded. The protein sequences obtained from blastp were than analyzed by use of NCBI Conserved Domain Search (http://www.ncbi.nlm.nih.gov/Structure/cdd/wrpsb.cgi) (Marchler-Bauer et al. 2005) for presence of conserved domains. Additionally, InParanoid7 (http://inparanoid.sbc.su.se/) was used to verify orthology of eukaryotic sequences (Ostlund et al. 2010).

The protein sequences of *C. merolae* were obtained from ‘*C. merolae* Genome Project’ (http://merolae.biol.s.u-tokyo.ac.jp). Sequences of *T. pseudonana*, *P. tricornutum*, *S. moellendorffii*, *E. huxlei*, *P. sojae, A. anophagefferens, M. brevicollis* proteins were acquired from Joint Genome Institute sequencing projects (http://genome.jgi-psf.org). The protein sequences from *D. discoideum* were obtained from dictyBase (http://dictybase.org). Sequences of proteins from *T. cruzi* were obtained from GeneDB (http://www.genedb.org). Sequences from other organisms were retrieved from GeneBank (http://blast.ncbi.nlm.nih.gov/Blast.cgi).

### Analysis of DNA and protein sequences

Target peptides were sought within amino acid sequences upstream of conserved catalytical domains of proteins of interest. To identify N-terminal extensions from the conserved regions of the proteins, sequences were analyzed with use of NCBI Conserved Domain Search (http://www.ncbi.nlm.nih.gov/Structure/cdd/wrpsb.cgi) (Marchler-Bauer et al. 2005). Prediction of subcellular localization was performed with TargetP 1.1 online software (http://www.cbs.dtu.dk/services/TargetP/). The analysis of sequences longer than 15 amino acids for presence of N-terminal motifs was performed as described in Emanuelsson et al. (2007). Settings were as follows: non plant organisms; prediction of cleavage site and without cut-offs. The output contains final Neuronal Networks scores (NN) (Nielsen et al. 1997) on which the prediction is based. The scores are not probabilities. However, the location with the highest score is the most likely according to TargetP. Each prediction has its reliability class (RC) expressed in number from 1 to 5, where 1 indicates the strongest prediction. Results of the analysis as well as conserved domain starting position are presented in Table 1. Presence of ER retention motifs in analyzed sequences (KDEL, DEEL or DDEL) was verified manually.

The detection of conserved motifs in DNA sequences of the up-stream regions of the *T. pseudonana* methionine metabolism genes was performed using an online tool MEME (http://meme-suite.org/meme/). The motif search was defined by following parameters: three best results, expected length 6–12 nt, occurrence once or none, ten sequences as a negative set. The sequences used in the analysis were obtained from *T. pseudonana* genome repository (http://genome.jgi-psf.org/Thaps3/Thaps3.home.html). The length of the DNA promoter region sequence of a gene was chosen to be longer than the half of a region between two ORFs (including UTRs). 5′-UTR, as defined by the repository, was included in the input data. In cases of longer stretches of DNA between genes 1,000 nt-long sequences were retrieved. The lengths, not including UTRs, are given in Table 2. The negative set, which served to verify the MEME results, composed of 5′-regions (500 nt + 5′-UTR) of ten non-sulfur-related proteins: actin-related protein, nitrate reductase, GAPDH, HSF60, citrate synthase, LHCr1, galactose kinase, MAP kinase, ubiquitin, tubulin, lipase. The results of the MEME analysis were applied to TOMTOM (http://meme-suite.org/tools/tomtom), which is the online software performing comparison of given motifs with available databases. Comparisons with plant (Jaspar 2014), prokaryotic (Prodoric 8.9) and yeast (MacIsaak v.1) databases were performed using default settings. The sequence-logo graphics was generated by the TOMTOM.

### Phylogenetic analysis

Phylogenetic trees were inferred using Phylogeny.fr (http://www.phylogeny.fr)—an on-line software package for sequence analysis (Dereeper et al. 2008). Sequences were aligned with MUSCLE 3.7 (Edgar 2004) and curation of the alignments was done with Gblocks 0.92 (Castresana 2000) using following settings:(a) conserved position: in at least half the number of sequences + 1; (b) flank position: at least 85% of the number of sequences; (c) maximum number of contiguous nonconserved positions: 8; (d) 10 amino acids as minimum length of a block; (e) now gaps allowed. The phylogenetic trees were reconstructed using the maximum likelihood method implemented in the PhyML program (v3.0 aLRT) (Guindon and Gascuel 2003). WAG substitution method was used as default. The bootstrapping procedure was replaced by an approximate likelihood-ratio test (aLRT) (Anisimova and Gascuel 2006). The test’s accuracy and power in comparison to the bootstrapping was discussed by Anisimova et al. (2011). Support values are presented at the nodes with a good statistics threshold set to 0.8. FigTree 1.3.1 software (http://tree.bio.ed.ac.uk/software/figtree/) was used to generate graphics.
